# Tracheal Intubation Without Use of Muscle Relaxants: Comparison of Remifentanil and Alfentanil

**DOI:** 10.5812/kowsar.22287523.3599

**Published:** 2012-01-01

**Authors:** Volkan Hanci

**Affiliations:** 1 Department of Anesthesiology and Reanimation, School of Medicine, Canakkale Onsekiz Mart University, Canakkale, Turkey

**Keywords:** Remifentanil, Alfentanil, Intubation

Dear Editor,

My objective is to analyze the efficiency of remifentanil-propofol and alfentanil-propofol combinations for laryngoscopic endotracheal intubation in the absence of muscle relaxants. This analysis reviews the work by Imani et al. (1). Muscle relaxants are frequently used to facilitate endotracheal intubation during anesthesia induction. However, the administration of short-acting depolarizing muscle relaxants is closely related to postoperative myalgias, malignant hyperthermia, hyperkalemia, and increased intracranial or intraocular pressure. Using non-depolarizing muscle relaxants may produce prolonged neuromuscular blockade, potentiate histamine release, increase the side effects from anticholinesterases used for reversing the agents, and lead to an inability to quickly reverse the blockade in the event of an unexpected difficult intubation. When using muscle relaxants is undesirable or contraindicated, it is important to administer other, proper induction agents to provide good intubating conditions (1, 2). Studies have investigated the use of propofol alone and propofol combined with other drugs (usually fentanyl, alfentanil, or remifentanil) for intubation without using a neuromuscular blockade; the findings have shown that intubation with all of these methods were successful (1-3). In another study, a dose of 2.5 mg/kg of propofol was applied without the use of a neuromuscular blockade, but only 20% of the patients had sufficient intubation scores (4). To decrease the necessary propofol dosage, eliminate unintented effects and to increase the potential of propofol’s effectiveness, propofol is often used together with opioids for intubation without a neuromuscular blockade. It has been reported that intubation scores were insufficient in the 17% of patients who were given fentanyl (1, 5). Alfentanil combined with propofol for intubation without a neuromuscular blockade may cause muscle rigidity and, especially at high doses, may lead to cardiovascular depression. Additionally, prolonging the effect of alfentanil after short-term operations is also undesirable (5, 6). Remifentanil is a phenyl-piperidine derivative that was first introduced into clinical practice in 1996. Remifentanil is 20 to 30 times more potent than alfentanil and its elimination half life is 3.8-8.3 minutes. Compared to alfentanil, remifentanil’s effect reduces much more quickly after intubation; this is an important advantage over alfentanil, especially in short-term and outpatient surgeries (1, 3, 6).

However, studies on propofol-alfentanil and propofol-remifentanil combinations are limited. Alexander et al. (7) compared the use of remifentanil (2 µg/kg), alfentanil (50 µg/kg), and succinylcholine (1 mg/kg) for intubation and found that perfect intubation scores were present in 35%, 85%, and 100% of the patients, respectively. The authors emphasized that remifentanil was an unsuitable option for intubation without a neumuscular blockade. Using a child sample, another study compared the effects of 4 mg/kg of propofol and 0.2 mg/kg of lidocain with 15 µg/kg of alfentanil or 1 µg/kg of remifentanil in intubation without a neuromuscular blockade. The findings revealed that neither the alfentanil nor remifentanil group caused a significant difference in intubation conditions (8). In a related study, Mohammadreza et al. (9) compared the effects of 5 mg/kg of thiopental with 40 µg/kg of alfentanil, 2 µg/kg of remifentanil, 3 µg/kg of remifentanil, and 4 µg/kg of remifentanil on intubation without a neuromuscular blockade. The authors reported that the best intubation conditions were available with 4 µg/kg of remifentanil and a combination of 40 µg/kg of alfentanil with 5 mg/kg of thiopental. Similarly, Klemola et al. (6) compared the effects of 2.5 mg/kg of propofol with 30 µg/kg of alfentanil, 3 µg/kg of remifentanil, and 4 µg/kg of remifentanilin on intubation without a neuromuscular blockade. The authors reported that the best intubation conditions were given with the combination of 4 µg/kg of remifentanil and 2.5 mg/kg of propofol. In another similar study, Erhan ve ark (9) compared the effects of 2 mg/kg of propofol with 40 µg/kg of alfentanil or 1, 2, 3, and 4 µg/kg of remifentanil on intubation without a neuromuscular blockade. The authors reported that the best intubation conditions were available with a combination of 4 µg/kg of remifentanil with 2 mg/kg of propofol. 

In a related study, Imani et al. (1) reported better intubation conditions with the combination of 5 µg/kg of remifentanil with 2 mg/kg of propofol than with 2 mg/kg of propofol, 50 µg/kg alfentanil, and 5 µg/kg of remifentanil. Nonhomogeneous distribution of doses, administration speed of agents, injection durations, time elapsed between injection and intubation, different scoring systems used for evaluation of intubation conditions, and age intervals of the cases enrolled in the studies all lead to difficulties in comparing these studies (1, 6-10). Some limitations are also present with Imani et al.’s study. First, they did not use a control group that received only propofol. In addition, the doses of remifentanil and alfentanil used in Imani et al.’s study are higher than in similar previous studies (6-10). As a result, the remifentanil-propofol combination seems to have advantages over the alfentanil-propofol combination because of the fast and short effect time of remifentanil. Nevertheless, evidence-based medical analyses, meta-analyses, and experimental studies with higher patient numbers are needed in this area.
